# Intake of l-serine before bedtime prevents the delay of the circadian phase in real life

**DOI:** 10.1186/s40101-022-00306-z

**Published:** 2022-08-26

**Authors:** Michihiro Ohashi, Sang-il Lee, Taisuke Eto, Nobuo Uotsu, Chie Tarumizu, Sayuri Matsuoka, Shinobu Yasuo, Shigekazu Higuchi

**Affiliations:** 1 Department of Kansei Science, Graduate School of Integrated Frontier Sciences, Kyushu University, 4-9-1 Shiobaru, Minami-ku, Fukuoka, 815-8540 Japan; 2 Research Fellow of the Japan Society for the Promotion of Science, 4-9-1 Shiobaru, Minami-ku, Fukuoka, 815-8540 Japan; 3 Laboratory of Environmental Ergonomics, Faculty of Engineering, Hokkaido University, Kita 13, Nishi 8, Kita-ku, Sapporo, Hokkaido 060-8628 Japan; 4 Department of Human Science, Faculty of Design, Kyushu University, 4-9-1 Shiobaru, Minami-ku, Fukuoka, 815-8540 Japan; 5 Research Institute, FANCL Co., 12-13, Kamishinano, Totsuka-ku, Yokohama, Kanagawa 244-0806 Japan; 6 Laboratory of Regulation in Metabolism and Behavior, Faculty of Agriculture, Kyushu University, 744 Motooka, Nishi-ku, Fukuoka, 819-0395 Japan

**Keywords:** Circadian rhythm, Sleep, l-Serine, Amino acids, University students, Field study

## Abstract

**Background:**

It has been shown in laboratory experiments using human subjects that ingestion of the non-essential amino acid l-serine before bedtime enhances the advance of circadian phase induced by light exposure the next morning. In the present study, we tested the effect of ingestion of l-serine before bedtime on *circadian phase* in real life and whether its effect depends on the initial circadian phase.

**Methods:**

The subjects were 33 healthy male and female university students and they were divided into an l-serine group (*n* = 16) and a placebo group (*n* = 17). This study was conducted in a double-blind manner in autumn and winter. After a baseline period for 1 week, the subjects took 3.0 g of l-serine or a placebo 30 min before bedtime for 2 weeks. Saliva was collected twice a week at home every hour under a dim light condition from 20:00 to 1 h after habitual bedtime. Dim light melatonin onset (DLMO) was used as an index of phase of the circadian rhythm.

**Results:**

DLMO after intervention was significantly delayed compared to the baseline DLMO in the placebo group (*p* = 0.02) but not in the l-serine group. There was a significant difference in the amount of changes in DLMO between the two groups (*p* = 0.04). There were no significant changes in sleeping habits after intervention in the two groups. There were significant positive correlations between advance of DLMO and DLMO before intervention in the l-serine group (*r* = 0.53, *p* < 0.05) and the placebo group (*r* = 0.69, *p* < 0.01). There was no significant difference in the slopes of regression lines between the two groups (*p* = 0.71), but the intercept in the l-serine group was significantly higher than that in the placebo group (*p* < 0.01). The levels of light exposure were not significantly different between the two groups.

**Conclusions:**

Our findings suggest that intake of l-serine before bedtime for multiple days might attenuate the circadian phase delay in the real world and that this effect does not depend on the initial circadian phase.

**Trial registration:**

This study is registered with University Hospital Medical Information Network in Japan (UMIN000024435. Registered on October 17, 2016).

## Background

The intrinsic period of the human circadian rhythm is longer than 24 h, and morning light plays an important role in resetting the circadian rhythm to 24 h [1, 2]. On the other hand, light at night from artificial lighting causes delays in sleep timing and circadian rhythm phases through non-visual effects of light [3–5]. In modern society, circadian phase delays can also be caused by nighttime digital media use [6, 7] and night work [8]. Since disruption of circadian rhythms due to these factors results in various negative health outcomes including insomnia, mood disorders, obesity, and type 2 diabetes, some countermeasures are needed [8, 9].

l-Serine is a non-essential amino acid synthesized in the body. It is also a precursor for the synthesis of other amino acids including the optical isomers D-serine and glycine as well as lipids and nucleotides [10] and it plays a role in mediating the nutritional supply for neuroglial cells [11, 12]. Furthermore, recent studies have shown that l-serine is involved in improvement of depression [13], Alzheimer’s disease [14], and *amyotrophic lateral sclerosis (ALS)* [15]. Those studies indicate that both l-serine biosynthesis and external ingestion are important for biological functions. Moreover, a previous study in which sleep quality was evaluated by using wrist actigraphy and self-reporting questionnaires showed that l-serine administration improved the subjective feeling of sleep and tended to decrease the number of nighttime awakenings *in humans* [16].

The results showing improved sleep quality in that previous study might be due to a contribution of l-serine to circadian entrainment. Our previous study [17] using mice and *healthy human subjects* showed that light exposure after administration of l-serine affected the phase shift of circadian rhythms. In the experiments using mice, we found that light-induced wheel-running phase shifts in mice that were administrated l-serine were greater than those in mice administrated water.

In a double-blind crossover test using twenty-one human subjects, subjects with a single intake of l-serine (3.0 g) before bedtime showed a significant circadian phase advance by bright light exposure in the morning (2000 lx, 90 min) compared with that in subjects in the placebo condition. The findings in our laboratory study indicate that l-serine may have the potential to promote light-induced circadian phase advance in humans. However, it was not clear whether the same results could be obtained in real life.

Therefore, in this study, we investigated whether l-serine intake before bedtime promotes circadian phase advance in real life. Since there are large individual differences in the circadian phase in humans in real life [18, 19], we also examined whether the effect of l-serine depends on the individual initial phase of the subject.

## Methods

### Participants and protocol

This study was conducted in Japan (Fukuoka City) in October and December. Thirty-three healthy university students participated in this study. None of the subjects were engaged in shift work or night shifts during the experimental period. We confirmed that melatonin secretion in all subjects started before the usual bedtime. The subjects were divided into two groups: a placebo group (13 men and 4 women, 22.1 ± 2.5 years old) and an l-serine group (10 men and 6 women, 21.3 ± 1.9 years old). Table 1 shows the characteristics of subjects in each group. All of subjects were Asian living in Fukuoka City, Japan. Chronotype was determined by the Japanese version of Morningness-Eveningness Questionnaire (MEQ) [20, 21]. There was no significant difference in sex ratio, age, or chronotype between the two groups. In addition, subjects answered their sleep habits on the Japanese version of the Munich ChronoType Questionnaire (MCTQ) [22] on their school days and free days. No significant differences were found between the two groups about all parameters of sleep habits. This study was carried out with the approval of the Ethics Committee of Kyushu University. The subjects were given oral and written explanations, and written consent for participation in the study was obtained.

**Table 1:** Characteristics of subjects in each group

	Placebo	l-serine	*p* value
	(*n* = 17)	(*n* = 16)	
Mean age [years]	22.1 ± 2.5	21.3 ± 1.9	*p* = 0.30
Sex	13M 4F	10M 6F	*p* = 0.42
MEQ score	48.1 ± 7.3	47.4 ± 8.5	*p* = 0.82
**Sleep habits (school days)**			
Bedtime	0:56 ± 0:53	0:36 ± 0:51	*p* = 0.29
Sleep latency [min]	16.9 ± 11.6	15.3 ± 8.5	*p* = 0.65
Sleep onset time	1:13 ± 0:58	0:51 ± 0:51	*p* = 0.27
Wake time	8:12 ± 0:54	8:01 ± 1:04	*p* = 0.59
Midpoint of sleep	4:42 ± 0:51	4:26 ± 0:50	*p* = 0.36
Sleep period time [h]	7.00 ± 0.77	7.16 ± 0.98	*p* = 0.60
**Sleep habits (free days)**			
Bedtime	1:18 ± 1:12	1:23 ± 0:47	*p* = 0.83
Sleep latency [min]	17.2 ± 12.2	15.3 ± 8.5	*p* = 0.60
Sleep onset time	1:35 ± 1:18	1:38 ± 0:50	*p* = 0.91
Wake time	9:11 ± 1:24	9:03 ± 0:55	*p* = 0.74
Midpoint of sleep	5:23 ± 1:17	5:20 ± 0:47	*p* = 0.90
Sleep period time [h]	7.59 ± 0.83	7.41 ± 0.82	*p* = 0.54
**Social jetlag [h]**	0.68 ± 0.68	0.90 ± 0.75	*p* = 0.37

Values are means and S.D

The experiment was conducted in a double-blind manner. The experiment consisted of a 1-week baseline period and a 2-week intervention period. After the start of the intervention, the subjects in each group ingested l-serine (3.0 g) or a placebo (Trehalose, 3.0 g) dissolved in 100 ml of water 30 min before bedtime every day. The intake dose was the same as that used in a previous study [16, 17]. The subjects were asked to keep their habitual sleep-wake schedule during the experiment. Excessive alcohol drinking and ingestion of sleeping pills were prohibited. The subjects were asked to record their sleep diary and wear a wrist actigraph device (MotionWatch 8, CamNtech, Cambridge, UK) for 24 h to determine their daily sleep-wake schedule and daily light exposure. The average illuminance of light exposure was log-transformed and calculated 5 h after the average wake time and 5 h before the average bedtime before and during the intervention period.

Saliva samples for melatonin assays were collected by each subject at home twice a week (on Thursday and Sunday). The subjects were forbidden from ingesting caffeine and performing strenuous exercise on the day of saliva collection. Saliva samples were collected every hour from 20:00 to each subject’s bedtime using a cotton swab (Salivatte® Sarstedt, Germany) under dim light in their home. The subjects were instructed to use an incandescent light bulb as indirect lighting from 19:30 in the room to make dim light (< 15 lx as measured by MotionWatch8). The subjects were also instructed to send a text message to the experimenter immediately after saliva collection.

Melatonin concentration in saliva was quantified by a radioimmunoassay (Buhlmann, RK-DSM2, Switzerland). Dim light melatonin onset (DLMO), a reliable marker of circadian phase [23, 24], was determined by linear interpolation between the time points before and after the melatonin concentration increased and stayed above the 3.0 pg/mL threshold [25].

### Statistical analysis

One subject who showed an irregular sleep/wake schedule was excluded from the analysis. Even after exclusion, there was still no significant difference in sex ratio, age, chronotype or sleep habits between the two groups. Another subject whose wearing time of the actigraph device during the day was too short for accurate determination of light exposure was also excluded from the analysis.

In the statistical analysis, a two-sided, Welch’s *t* test was used for comparison of sleep habits, DLMO, amount of DLMO advance, and light exposure between the two groups. A two-sided paired *t* test was used for investigating changes in sleep habits and DLMO within the groups. Greenhouse–Geisser correction was performed whenever Mauchly’s test of sphericity was significant. Pearson’s correlation test was used to assess the relation between DLMO before intervention and DLMO advance in each group. Afterwards, the general linear model (GLM) was used for comparison of the slopes and intercepts of regression lines between the two groups. A *p* value less than 0.05 was considered statistically significant. Statistical analyses were performed using SPSS software ver. 25 (IBM, NY, USA).

## Results

**Fig. 1:**
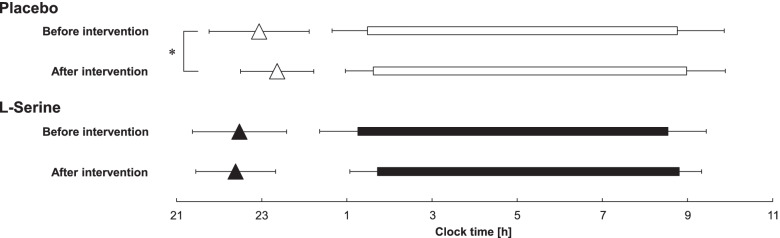
DLMO and sleep/wake time before and after intervention in each group (mean ± S.D., *: *p* < 0.05) (The triangles represent DLMO and the thick horizontal bars represent bedtime to wake time)

There were no significant differences in bedtime (*p* = 0.47), wake time (*p* = 0.55), time in bed (*p* = 0.98), and DLMO (*p* = 0.26) between the two groups before intervention (Table 2 and Fig. 1). A significant difference before and after intervention was found for DLMO in the placebo group but not in the l-serine group (Table 2). DLMO in the placebo group was significantly delayed after intervention (*t* = − 2.59, *p* < 0.05). No significant changes in sleep measurements after intervention were found in the two groups.

**Table 2:** DLMO and sleep/wake time before and after intervention in each group

	Placebo(*n* = 17)			l-serine(*n* = 15)			*p* value (Before intervention, between groups)
	Before intervention	After intervention	*p* value (before vs. after)	Before intervention	After intervention	*p* value (before vs. after)	
**DLMO**	22:56 ± 1:11	23:22 ± 0:52	*p* = 0.02*	22:29 ± 1:06	22:23 ± 0:56	*p* = 0.62	*p* = 0.26
**Bedtime**	1:29 ± 0:50	1:37 ± 0:39	*p* = 0.21	1:16 ± 0:54	1:43 ± 0:39	*p* = 0.13	*p* = 0.47
**Wake time**	8:45 ± 1:06	8:59 ± 0:55	*p* = 0.16	8:32 ± 0:54	8:48 ± 0:32	*p* = 0.30	*p* = 0.55
**Time in bed [h]**	7.27 ± 0.86	7.36 ± 0.71	*p* = 0.53	7.28 ± 0.61	7.09 ± 0.73	*p* = 0.24	*p* = 0.98

Values are means and S.D

*p < 0.05

**Fig. 2:**
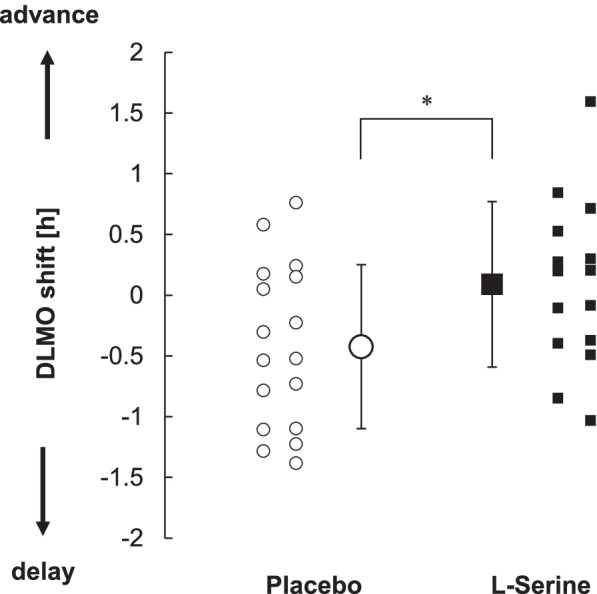
Individual data for amount of DLMO advance (mean ± S.D., *: *p* < 0.05)

The magnitudes of changes after intervention were compared in the placebo group and l-serine group (Table 3). A significant difference in changes in DLMO was found between the two groups (placebo < l-serine, *t* = − 2.14, *p* = 0.04) (Table 3 and Fig. 2).

**Table 3:** Amount of advance in DLMO and sleep/wake time in each group

	Placebo (*n* = 17)	l-serine (*n* = 15)	*p* value
**DLMO**	− 0:25 ± 0:41	0:05 ± 0:41	*p* = 0.04*
**Bedtime**	− 0:08 ± 0:26	− 0:28 ± 1:06	*p* = 0.30
**Wake time**	− 0:13 ± 0:37	− 0:16 ± 0:57	*p* = 0.88
**Time in bed [h]**	0.09 ± 0.54	− 0.19 ± 0.61	*p* = 0.18

Values are means and S.D. *p < 0.05

Amount of increase only in time in bed

**Fig. 3:**
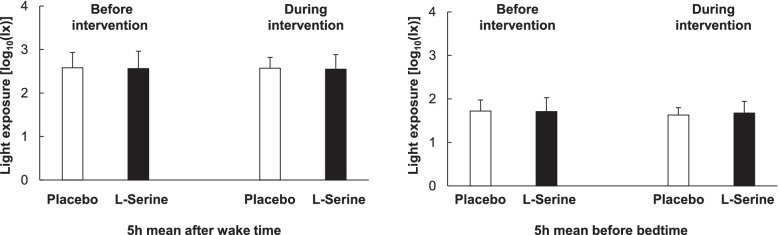
Five-hour mean light exposure after wake time and before bedtime (mean ± S.D.)

Figure 3 shows the results for mean light exposure. No significant differences were found after wake time before intervention (*p* = 0.89), after wake time during intervention (*p* = 0.84), before bedtime before intervention (*p* = 0.90), and before bedtime during intervention (*p* = 0.58).

**Fig. 4:**
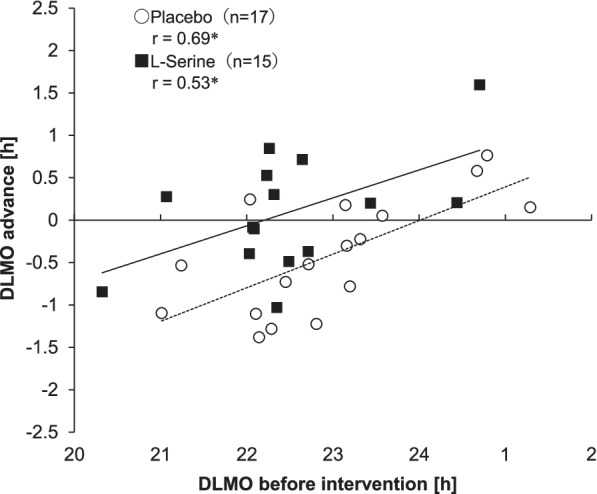
Relationship between DLMO before intervention and DLMO advance (*: *p* < 0.05)

Figure 4 shows the relationship between DLMO before intervention and DLMO advance. There were significant positive correlations between the advance of DLMO and DLMO before intervention in the l-serine group (*r* = 0.53, *p* < 0.05) and the placebo group (*r* = 0.69, *p* < 0.01).

The GLM with groups as a dependent variable and DLMO before intervention as a between-subject factor was used to test independence of DLMO before intervention. The main effect of groups was not statistically significant (*p* = 0.26), and independence of DLMO before intervention was thus confirmed. The GLM with DLMO advance as a dependent variable, groups as a between-subject factor and DLMO before intervention as a covariate was used to compare the slopes of regression lines. The interaction between groups and DLMO before intervention was not statistically significant (*p* = 0.71). Therefore, it was confirmed that there was no significant difference in slopes of regression lines. The GLM without interaction between groups and DLMO before intervention was used to test the significance of regression and to compare the intercepts of regression lines. The effect of DLMO before intervention was statistically significant (*p* < 0.001), and the significance of regression was therefore confirmed. Moreover, the main effect of groups was statistically significant (η_p_^2^ = 0.30, *p* < 0.01). This means that the intercept of the regression line in the l-serine group was significantly greater than that in the placebo group.

## Discussion

In this study, a significant delay in DLMO was confirmed in the placebo group but not in the l-serine group. Moreover, the amount of DLMO changes was significantly different between the two groups. However, given that there were no significant differences between the groups in sex, age, MEQ score, and results for DLMO and sleep habits before the intervention, it is difficult to interpret that the characteristics of the group caused the result of phase delay in the placebo group. Furthermore, the amounts of exposure to daily light just before bedtime and after wake time, which are thought to affect the results of DLMO shift, were not significantly different between the two groups.

In the present study, we showed that l-serine has a preventative effect on the delay of circadian rhythms. However, unlike in the laboratory experiments conducted in our previous study [17], no significant phase advance of the circadian rhythm was observed by l-serine intake before bedtime. One possible reason for this may be the difference in the light environment between the laboratory and field experiments. In the laboratory experiment, evening light (from 6 pm to bedtime) was dim light (< 15 lx), whereas in the field experiment, the subjects were exposed to night light by artificial lighting except on days when saliva samples for melatonin measurement were taken. It is known that artificial lighting at night in real life delays the phase of circadian rhythms [26, 27]. In other words, the lack of phase advance in this study may have been canceled out by phase delay due to exposure to light at night.

Thus, if the effects of night light cannot be avoided in daily life, increasing l-serine intake might be a possible way to induce the phase advance that was observed in the laboratory experiments [17]. However, while a dose-response relationship was observed for the effects of l-serine in a previous animal study [17], no comparison of the effects of increased intake in humans has been conducted. For example, patients with ALS were tested with l-serine at doses of up to 15 g twice daily for 6 months and no serious adverse effects were observed and the drug is generally considered safe [15]. Since l-serine is “generally recognized as safe” by the Food and Drug Administration (FDA), field studies should be conducted in the future to examine the effects of increased doses of l-serine on circadian rhythm phase advance.

In addition to eating behavior, exercise habits have a potential to impact on light-induced circadian phase shift [28]. In many studies, physical activity level was measured by using a wrist accelerometer [29, 30]. However, in the present study, wrist accelerometer data were measured to mainly determine the daily sleep-wake timing and daily light exposure of the subjects and we instructed the subjects to remove the wrist accelerometer during intense exercise to avoid damage to the device. Determination of the interactions between light exposure and physical activity including their timing and intensity in terms of circadian phase shift in daily life would be challenging but is expected to be done in a future study.

We did not expect the delay in DLMO observed in the placebo group. This result means that the phase of the circadian rhythm was delayed in the placebo group, and there could be several reasons for this. First, in the period of October to December, when this experiment was conducted, the time of sunrise is becoming later and the amount of sunshine is less than that in summer in the Northern Hemisphere. It is well known that the light and dark cycle of sunlight is the strongest zeitgeber for circadian rhythm and that morning light is important to reset the human circadian period, which is intrinsically longer than 24 h [1]. Some studies showed that circadian phase in winter was later than that in summer in countries in the Northern Hemisphere [31–33]. Next, the subjects in this study were healthy university students. University students generally tend to be night owls biologically and socially [34, 35] and experience relatively little light exposure in the morning and more light exposure later in the day in terms of both intensity and duration [36, 37]. It is possible that these factors affected the easiness for delay of circadian phase in daily life.

In this study, trehalose was used as a placebo. Although animal studies suggested that trehalose has effects in neurodegenerative diseases such as Huntington’s disease [38], Alzheimer’s disease [39], and ALS [40], there has been no study in which the relationship between trehalose ingestion and circadian rhythms was investigated in animals and humans. On the other hand, it has been reported that carbohydrate intake is related to sleep and circadian rhythms in humans through blood glucose levels [41, 42]. In addition, animal studies have shown that insulin directly affects the peripheral circadian clock [43, 44]. Although trehalose is converted to glucose and increases insulin concentrations [45], the dose of trehalose used as a placebo in this study (3.0 g) was very small, and its effect on glucose and insulin concentrations is likely to be limited. Therefore, the possibility that the placebo condition directly affected the delay in circadian rhythm is considered negligible. However, since we did not examine or control for the subjects’ daily diet in this experiment, we cannot exclude the possibility that differences in eating behavior between the two groups may have affected the results.

In this study, the regression coefficient by DLMO before intervention was significant for the amount of DLMO advance. A significant positive correlation between advance of DLMO and DLMO before intervention was found in both groups. This indicates that the later DLMO was, the greater was the subsequent DLMO advance. One probable cause of this is that the phase angle between the circadian phase and sleep timing affected the amount of circadian phase shift. When sleep timing is primarily tied to social factors, late circadian timing results in a short phase angle. If the phase angle is short, the phase delay zone around DLMO of the phase response curve (PRC) to light [46, 47] is masked by sleep. It is thought that the circadian phase tends to advance thereafter.

On the other hand, the effect of the group on DLMO before intervention was not significant, and there was no difference in DLMO before intervention between the two groups and DLMO before intervention did not depend on the group. Uniformity of the slopes of regression lines in the two groups was also shown. In addition, a significant difference in the intercepts of regression lines was found between the two groups. These results suggest that the DLMO advance in the l-serine group was greater than that in the placebo group in all ranges of confirmed DLMO in this study. The fact that the effect of l-serine was confirmed regardless of the subject's circadian phase means that l-serine may be useful for various chronotypes of people.

In recent years, much attention has been focused on research on the relationship between social jetlag and health [48]. In the present study, the effects of social jetlag did not be assessed because the average social jetlag of the subjects was less than 1 h (Table 1). This small social jetlag may be related to the less social constraints of weekday life for university students. However, it is known that social jet lag is greater in nocturnal chronotypes [49] and that loss of morning light exposure due to weekend recovery sleep leads to a delay of the circadian phase [50]. The effects of l-serine in populations with greater social jetlag should be examined in future studies.

The effect of l-serine was confirmed in an autumn/winter experiment, but it is necessary to consider seasonal differences in the effect of l-serine in consideration of the day length and amount of daytime sunshine in various seasons. However, considering that seasonal affective disorder [51], which is presumed to be associated with circadian rhythm disorder, is likely to develop in the period in which this study was conducted and considering the results of a study showing that l-serine enhanced the antidepressant effect of light in a mouse model of seasonal affective disorder [52], the effect of l-serine on light-induced circadian phase advance in humans that was confirmed in this study is a notable finding. This study was conducted in healthy subjects, and the effectiveness of l-serine in patients with circadian rhythm disorders as well as patients with seasonal affective disorder should also be examined.

Finally, although the mechanism by which l-serine affects the light-induced phase shift of circadian rhythms is not yet well understood, our previous study in mice (CBA/N) showed that light exposure after l-serine ingestion altered the long-term expression pattern of clock genes in the suprachiasmatic nucleus (SCN) [17]. As a possible mechanism for this, we focused on the MNDA receptor, which is believed to have a major role in light signaling in the SCN [53]. However, since the effectiveness of l-serine was not blocked by MK801, an antagonist of the NMDA receptor, we considered that this receptor was not involved [17]. On the other hand, we found that antagonists of the GABA_A_ receptor completely blocked the effectiveness of l-serine. In other words, l-serine was thought to affect circadian rhythm phases via activation of GABA_A_ receptors. This is consistent with the results of a previous study showing that the sedative and hypnotic effects of l-serine are mediated by GABA_A_ receptors [54]. GABA_A_ is also known to play an important role in circadian clock function in the SCN [55]. Therefore, the light-induced phase advance of l-serine in mice may be involved in the phase shift of circadian rhythms via the GABAergic system. Furthermore, l-serine did not affect the light-induced expression of c-fos, Per1, and Per2 in the SCN, but it altered the long-term expression of Per2 and Bmal1 [17]. These results suggest that the action of l-serine occurs in extra-SCN regions that convey information to the SCN via a post-transcriptional process. However, given that the mechanism of effects of light may differ among species [17], it is unclear whether the same mechanism of action is also true in humans. In addition, there are limitations in referring to the mechanism based on the results obtained in the present field study.

## Conclusions

The results of this study showed that external intake of l-serine before bedtime for multiple days might enhance circadian phase advance by light in the morning and suppress the circadian phase delay. The results also suggested that the effect of l-serine does not depend on the initial circadian phase. l-serine intake before bedtime is expected to help prevent the delay of circadian rhythm in real word conditions in various chronotypes of people.
